# Ureteroscopy for stone disease in the elderly (≥ 80 years): Outcomes of a multicentre study from YAU and EAU endourology groups

**DOI:** 10.1007/s00240-025-01890-2

**Published:** 2025-11-18

**Authors:** Lazaros Tzelves, Bhaskar Somani, Steffi Kar Kei Yuen, Vineet Gauhar, Smita De, Amelia Pietropaolo, Daniele Castellani, Deepak Ragoori, Esteban Emiliani, Eugenio Ventimiglia, Arman Tsaturyan, Tarik Emre Sener, Maria Florencia Frascheri, Alba Sierra, Francesco Esperto, Łukasz Nowak, Vincent De Coninck, Ioannis Mykoniatis, Alberto Olivero, Rifat Burak Ergül, Begoña Ballesta Martinez, Alejandro Bautista-Perez-Gavilan, Sahil Patel, Eric Villalba, Qasim Seyidov, Joren Vanthoor, Stavros Tsiakaras, Panagiotis Triantafyllou, Arianna Pischetola, Luca Villa, Paola Arena, Øyvind Ulvik, Mathias S. Æsøy, Peder Gjengstø, Ali Talyshinskii, Christian Beisland, Patrick Juliebø-Jones

**Affiliations:** 1 2nd Department of Urology, Sismanoglio Hospital, National and Kapodistrian University of Athens, Athens, Greece; 2 EAU Young Academic Urologists (YAU) Urolithiasis and Endourology Working Group, Arnhem, The Netherlands; 3 EAU Endourology Endourology Section, Arnhem, The Netherlands; 4 Department of Urology, University Hospitals Southampton, NHS Trust, Southampton, UK; 5 Department of Surgery, SH Ho Urology Centre, The Chinese University of Hong Kong, Hong Kong, China; 6 Department of Urology, Ng Teng Fong General Hospital, Singapore, Singapore; 7 Department of Urology, Asian Institute of Nephrology and Urology, Hyderabad, India; 8 Department of Urology, Cleveland Clinic Glickman Urological & Kidney Institute, Cleveland, OH USA; 9 Urology Unit, Azienda Ospedaliero-Universitaria delle Marche, Ancona, Italy; 10 Department of Urology, Fundació Puigvert, Universitat Autonoma de Barcelona, Barcelona, Spain; 11 Department of Urology, NYU Langone Health, NYU Grossman School of Medicine, New York, USA; 12 Department of Surgical Sciences, Uppsala University, Uppsala, Sweden; 13 Department of Urology, Yerevan State Medical University after M. Heratsi, Yerevan, Armenia; 14 Department of Urology, Erebouni Medical Center, Yerevan, 0087 Armenia; 15 Department of Urology, School of Medicine, Marmara University, Istanbul, Turkey; 16 Department of Urology, Hospital Alemán de Buenos Aires, Buenos Aires, Argentina; 17 Department of Urology, Hospital Clínic de Barcelona - University of Barcelona, Barcelona, Spain; 18 Department of Urology, Campus Bio-Medico University, Rome, Italy; 19 Department of Minimally Invasive and Robotic Urology, University Center of Excellence in Urology, Wroclaw Medical University, Wroclaw, Poland; 20 Department of Urology, AZ Klina, Brasschaat, Belgium; 21 Department of Urology, Aristotle University of Thessaloniki, Thessaloniki, 541 24 Greece; 22 Department of Urology, ASST Grande Ospedale, Metropolitano, Milan, 20162 Niguarda Italy; 23 Department of Urology, Istanbul Faculty of Medicine, Istanbul University, Istanbul, Turkey; 24 Department of Urology, University Hospital Del Vinalopó, Alicante, Spain; 25 Division of Experimental Oncology/Unit of Urology, Urological Research Institute, IRCCS Ospedale San Raffaele, Milan, Italy; 26 Department of Clinical Medicine, University of Bergen, Bergen, Norway; 27 Department of Urology, Haukeland University Hospital, Bergen, Norway; 28 Astana Medical University, Astana, Kazakhstan

**Keywords:** Ureteroscopy, Elderly, Mortality, Complications, Older, Urolithiasis

## Abstract

The objective of this study was to perform a multi-centre and global study evaluating the clinical outcomes and complications associated with of URS for stone disease in patients aged 80 years and above. Retrospective analysis was conducted on patients aged ≥ 80 years who underwent URS for stone disease between January 2014 and December 2024, across 20 centres in 14 countries. A total of 679 patients aged ≥ 80 years underwent ureteroscopy (URS), with a median age of 83 years (IQR 81–86) and a male-to-female ratio of 1.2:1. Most patients were ASA grade 2 (52%) and presented electively (95%), with general anaesthesia used in 77% of cases. The median cumulative stone size was 11 mm (IQR 7–15), and 56% had a single stone. The overall stone-free rate (SFR) was 70% for zero fragments, increasing to 84% when including fragments ≤ 2 mm, and 90% when including fragments ≤ 4 mm. The overall 30-day complication rate was 16%, with major complications (Clavien-Dindo grade ≥ 3) in 2.8% of patients. The most common complication was urinary tract infection (9.4%). One procedure-related death (0.1%) was recorded. Multivariable analysis identified ASA score ≥ 3 (OR 1.60), operative time > 60 min (OR 1.73), and emergency surgery (OR 2.96) as independent predictors of complications. Ureteroscopy can be performed in patients aged 80 years and above with a low risk of major complications and acceptable stone clearance rates. Avoiding both emergency surgery and prolonged operative times can help reduce the morbidity profile.

## Introduction

Urolithiasis affects 1–20% of people worldwide, with a 50% recurrence rate over ten years [1]. Ureteroscopy (URS) is now the preferred surgical intervention for ureteral and kidney stones, with over 150,000 procedures performed annually in the US, making up around two-thirds of all stone treatments [2]. As global demographics shift toward an older population—such as in Japan and Italy where more than 21% are over 65—the incidence of stones peaks in the elderly [3]. However, most research targets younger patients, and older adults are often excluded from surgical studies, despite evidence that they have lower rates of spontaneous stone passage and require more interventions as was shown by Krambeck et al. [3]. International guidelines lack specific recommendations for elderly patients, particularly those over 80 years, despite Penniston et al. showing that it is the elderly age at which stone incidence can peak [4]. As evidence in URS for exclusively octogenarians and nonagenarians is limited to address this gap, we conducted a multi-centre study assessing outcomes of URS for patients aged ≥ 80 years with ureteral and/or kidney stone disease. To this end, our aim was to perform a multi-centre and global study evaluating the clinical outcomes and complications associated with of URS for stone disease in patients aged 80 years and above.

## Methods

### Data collection

A retrospective analysis was conducted on patients aged ≥ 80 years who underwent URS for stone disease between January 2014 and December 2024, across 20 centres in 14 countries including Asia, Europe and North America. Inclusion criteria encompassed patients aged ≥ 80 years, with kidney and/or ureteral stone(s) and deemed suitable for URS from the treating physician. URS was performed as per the standard of care and surgical practice of each contributing institution. Approval by the ethical or audit committee was gained in accordance with local regulations.

## Outcomes of interest

The primary outcome of this analysis was stone-free rate (SFR). The secondary outcome was the incidence of postoperative complications reported within 30 days of surgery. Complications were graded according to the modified Clavien Dindo (CD) system. SFR was categorized as follows: (A) zero fragments, (B) fragments ≤ 2 mm, (C) fragments 2.1–4 mm. Timing of imaging was based on local protocol. Data were also collected on baseline demographics, including American Society of Anesthesiologists (ASA) grade; operative details, such as the use of a ureteral access sheath (UAS); and follow-up information, including mortality status at six months postoperatively. The latter was available in all patients. Data were registered for only one procedure per patient, rather than multiple procedures for the same individual in case of the need of ancillary procedure(s) for residual fragment(s).

### Statistical analysis

All data was anonymised before being pooled at the coordinating centre. Continuous variables were described using median and interquartile range, while categorical variables were described using absolute numbers and percentages. Chi-square test was applied to compare categorical data, while Mann-Whitney U test for continuous variables. Multivariable logistic regression was performed to identify predictors of post-operative complications. These predictors were described using odds ratio (OR), 95% confidence interval (CI), and p-values. Statistical analysis was performed using SPSS version 29 (IBM Corporation, Armonk, NY, USA), with *p* < 0.05 determining statistical significance.

## Results

Overall, 679 patients underwent URS, with a median age of 83 years (interquartile range (IQR): 81–86) and a male-to-female ratio of 1.2:1. The majority of patients were ASA 2 (52%, *n* = 349) and had presented with pain (45%, *n* = 299). The median cumulative stone size was 11 mm (IQR 7–15) and most (56%, *n* = 382) had a single stone (Table 1). Non contrast computed tomography (NCCT) was the commonest imaging modality to assess stone burden in 95% (*n* = 637) of patients. Most cases were performed in an elective setting (95%, *n* = 642) and under general anaesthesia (77%, *n* = 525). The indications for emergency URS were all for patients admitted acutely with uncontrolled pain without evidence of infection. The median operative time was 58 min (IQR 40–83) with ureteral access sheath (UAS) being used in half of the cases (Table 2). Overall, 4% (*n* = 27) cases were not completed as planned due an intra-operative complication. For the latter, the most frequently reported was bleeding obscuring vision (1.5%, *n* = 10). Majority of patients (93%) received a ureteral stent postoperatively and the median hospital stay was 2 days (IQR 1–3). The overall post-operative complication rate was 16% (*n* = 108) (Table 3). Based on CD system for grading severity, the minor (CD grade up to 2) and major complication (CD grade ≥ 3) rate was 13.2% (*n* = 89) and 2.8% (*n* = 19), respectively. The most frequently recorded complications were urinary tract infection (CD grade 2) (9.4%, *n* = 64), haematuria requiring catheter irrigation (CD grade 2) (1.3%, *n* = 9) and haematuria managed conservatively (CD grade 1) (0.9%, *n* = 6). One death (CD grade 5) was recorded within 30 days, and this was determined to be procedure related (sepsis). No significant difference in overall postoperative complications was recorded among patients with ureteral stones (16.2%), kidney stones (14.8%), and stones in both locations (20.8%, *p* = 0.53). Emergency surgeries had a significantly higher risk of postoperative complications compared to elective surgeries (32.4% vs. 14.8%, odds ratio (OR) = 2.76, 95% confidence interval (CI): 1.3–5.7, *p* = 0.01). At 6 months postoperatively, 88% of patients were still alive.

**Table 1:** Summary of baseline dempgraphics

Sample size	679
Age (yr), median (IQR)	83 (81–86)
Male-female ratio	1.2:1
*ASA*	
Grade 1	22 (3.2%)
Grade 2	349 (52%)
Grade 3	279 (41%)
Grade 4	27 (4%)
Oral anticoagulant use	195 (29%)
Normal renal anatomy	641 (94%)
*Abnormal renal anatomy*	
Solitary kidney	21 (55%)
Urinary diversion	5 (13%)
Duplex system	4 (11%)
Atrophic kidney	3 (7.9%)
Horseshoe kidney	2 (5.3%)
Other	3 (7.9%)
*Presentation*	
Pain	299 (45%)
Sepsis	124 (19%)
Surveillance imaging	106 (16%)
Haematuria	48 (7.2%)
Acute kidney injury	62 (9.2%)
Recurrent infection	30 (4.5%)
Other	2 (0.3%)
*Stone location*	
Ureter	235 (35%)
Kidney	391 (58%)
Ureter + kidney	53 (7.8%)
*Number of stones*	
Single	382 (56%)
Multiple	297 (44%)
Cumulative size (mm), median (IQR)	11 (7–15)
Hounsfield units, median (IQR)	980 (702–1200)
*Imaging type*	
NCCT	637 (94%)
XR KUB	28 (4.1%)
USS	13 (1.9%)
Impacted stone	115 (17%)
*Setting*	
Elective	642 (95%)
Emergency	37 (5.4%)
Pre-stented	340 (50%)

**Table 2:** Summary of operative data

*Anaesthesia*	
General	525 (77%)
Spinal	147 (22%)
Sedation	7 (1%)
Operation time (mins), median (IQR)	58 (40–83)
Use of UAS	341 (50%)
Intraoperative complication causing operation to be terminated early	27 (4%)
*Intra-operative complication*	
Bleeding	10 (1.5%)
Ureter injury	4 (0.7%)
Sepsis	3 (0.4%)
Peri-renal haematoma	1 (0.15%)
Other	9 (1.3%)
Post-operative stent	628 (93%)
Hospital stay (days), median (IQR)	2 (1–3)

**Table 3:** Summary of follow up data

*Post operative complications*			
Overall rate	108 (16%)		
Minor (Grade I–II)	89 (13.2%)		
Major (≥ Grade III)	19 (2.8%)		
*Complication type*			
UTI	64 (9.4%) CD II		
Urinary sepsis	12 (1.8%) CD III		
Haematuria with return to theatre	2 (0.3%) CD III		
Haematuria with catheter irrigation/washout	9 (1.3%) CD II		
Haematuria – conservative	6 (0.9%) CD II		
Acute obstruction due to residual fragments	5 (0.7%) CD III		
Pain	4 (0.6%) CD II		
Aspiration pneumonia	2 (0.3%) CD II		
Peri-renal haematoma	1 (0.15%) CD II		
Hypotension	1 (0.15%) CD I		
Hypoxia	1 (0.15%) CD II		
Death	1 (0.15%) CD V		
*Follow up imaging*			
XR KUB	253 (37%)		
NCCT	215 (32%)		
USS	173 (26%)		
XR + USS	33 (4.9%)		
Missing data (did not attend imaging)	4 (0.6%)		
Time to follow up imaging (weeks), median (IQR)	12 (6–12)		
New stricture at follow up imaging	21 (3.1%)		
Alive at 6 months	599 (88%)		

Stone free rate	Overall	NCCT	Other imaging (ultrasound/x-ray)
*All Stones*			
Zero fragments	70%	63%	80%
2 mm or less	84%	79%	92%
2.1–4mm	90%	86%	93%
*Renal stones only*			
Zero fragments	67%	60%	71%
2 mm or less	85%	81%	87%
2.1–4mm	89%	87%	90%
*Ureteral stones only*			
Zero fragments	78%	72%	80%
2 mm or less	85%	79%	87%
2.1–4mm	91%	87%	94%
*Renal + Ureteral stones only*			
Zero fragments	51%	42%	59%
2 mm or less	72%	67%	76%
2.1–4mm	85%	79%	90%

Multivariable logistic regression revealed that ASA score ≥ 3 (OR) 1.60, 95% confidence interval (CI) 1.04–2.47; *p* = 0.03), operation time > 60 min (OR 1.73, 95% CI 1.13–2.66; *p* = 0.01) and emergency surgery (OR 2.96, 95% CI 1.40–6.25; *p* = 0.02) were factors associated with higher odds of having postoperative complications (Table 4).

**Table 4:** Multivariable logistic regression for predictors of postoperative complications

Variable	OR	95% CI	*p*-value
ASA ≥ 3	1.60	1.04–2.47	0.03
*Operation time*			
> 60 min	1.73	1.130–2.658	0.01
*Gender*			
Male	1.17	0.76–1.79	0.49
Female	–	–	–
*Emergency surgery*			
Yes	2.96	1.40–6.25	0.005
No	–	–	–
Age ≥ 85	0.88	0.57–1.38	0.59

Median time to follow up imaging was 12 weeks (IQR 6–12) and this was most commonly plain x-ray only (37%, *n* = 253) or NCCT (32%, *n* = 215).

SFR was as follows: (A) Zero fragments = 70%, (B) including fragments ≤ 2 mm = 84%, (C) including fragments ≤ 4 mm = 90%. The zero-fragment SFR varied significantly by stone location: ureter (78%), kidney (68%), and combined ureter and kidney (51%) (*p* < 0.001). Among patients with kidney stones (*n* = 391, 58%), the use of a UAS was not associated with improved SFR (72% without UAS vs. 66% with UAS, *p* = 0.255). A breakdown of SFR based on NCCT imaging vs. all other types as well as by location is provided in Table 3.

There was no significant difference in postoperative complication rates between procedures performed with and without UAS (14.5% vs. 15.5%, *p* = 0.0794). Pre-stenting also did not impact the SFR (68% vs. 68%, *p* = 0.965).

## Discussion

This large, multicentre retrospective study of patients aged 80 years and above demonstrated an acceptable rate of complications, with a low incidence of major postoperative adverse events. While the SFR for renal stones was modest at 51%, it is comparable to real-world data from the Michigan Urological Surgery Improvement Collaborative (MUSIC) registry, which reported a similar SFR of 49.6% regardless of patient age [5]. It is arguable that treatment objectives in this age demographic are subtly different to those for younger patients. In older individuals, the primary goal may shift toward alleviating the symptomatic burden of the index stone rather than achieving absolute stone-free status. Consequently, small, non-obstructing residual fragments may be considered more suitable for conservative management.

The retrospective design introduces inherent selection bias. Nonetheless, at six months post-procedure, over 10% of patients had died, but we did not gather the cause of death. Nevertheless, this mortality underscores the importance of carefully weighing the benefits of intervention against the risks and life expectancy in this population.

The role of UAS during URS continues to generate debate. In the present study, cases performed using suction ureteral access sheaths (FANS) were not included. This decision reflected the study period (January 2014–December 2024), during which FANS technology was not routinely available, and many participating centres still did not have access to it. We therefore did not collect data for procedures in which FANS had been used, to maintain consistency and comparability of outcomes across all centres. Recent evidence supports the premise that employment of the latter as well as direct in scope suction (DISS) could improve SFRs further [6–8]. Such is the continued development in these technologies as well as energy sources, the use of URS for urolithiasis in elderly appears likely to continue to rise as the intervention of choice. Alternative treatment options do include shockwave lithotripsy (SWL), which is less invasive; however, comorbidities commonly associated with advanced chronological age, such as dementia and use of antiplatelets/anticoagulants, often represent relative contraindications. Furthermore, previous studies have reported reduced stone clearance rates with increasing age [9, 10]. Similarly, age per se does not preclude percutaneous nephrolithotomy (PCNL). Several series have reported good outcomes in older populations [11, 12]. However, the higher morbidity burden traditionally associated with PCNL, albeit lower with miniaturised equipment, likely prompts surgeons to be more cautious in selecting this modality. Performing a single session of URS and then re-evaluating the need for a second operation once completed, may be considered a safer option but this approach should be balanced against the need for two sessions of anaesthesia that could be detrimental for comorbid patients. The choice of modality should also likely reflect what the surgical team is most experienced with. The higher intraoperative complication compared to index patients highlights additional factors to consider when performing such surgery. A key one reflected in this study is for instance bleeding from the prostate.

URS is now routinely performed as a day-case procedure in most centres for younger or otherwise fit patients. The median hospital stay of 2 days recorded in this study is therefore notable and likely reflects the characteristics of the elderly cohort and the protocols of participating hospitals. Contributing factors include the need for closer postoperative monitoring, a lower threshold among clinicians to extend observation before discharge, and social considerations such as the need to arrange additional home or caregiver support prior to discharge.

There are several limitations to acknowledge in this study. This includes the retrospective nature and the heterogeneity in terms of pre and post imaging types and absence of data on stone volume. Information regarding the cause of death and elective reoperation rates was not available. The differences in the choice of follow-up imaging reflect variations across healthcare systems, resource availability, and cost considerations among the participating centres. In a recent study evaluating imaging practices in endourology across 32 countries, the mean cost of a plain x-ray was €27.1 compared with €105.8 for a NCCT [13].

Nonetheless, it represents real world practice across three continents and adds reference data on a demographic that is increasing in size yet is still under researched. Future studies should adopt a prospective design with standardised follow-up protocols, ideally incorporating low dose NCCT imaging, to more accurately assess residual stone burden. The incorporation of validated frailty scoring systems and quality-of-life assessments in future studies would provide additional insight into functional outcomes and patient-centred aspects of care in this elderly cohort.

## Conclusion

Ureteroscopy can be performed in patients aged 80 years and above with a low risk of major complications and acceptable stone clearance rates. Avoiding both emergency surgery and prolonged operative times can help reduce the morbidity profile. The decision to operate requires careful consideration and made on a case-by-case basis.

## Data Availability

The data sets generated during and/or analyzed during the current study are available from the corresponding author on reasonable request.
